# Evaluation by MRI of left ventricular remodeling and global functional recovery in patients treated with granulocyte-colony stimulating factor (G-CSF) after acute myocardial infarction (AMI)

**DOI:** 10.1186/1532-429X-11-S1-P191

**Published:** 2009-01-28

**Authors:** Antonio Bernardini, Luigi Natale, Agostino Meduri, Carlo Liguori, Antonio Maria Leone, Lorenzo Bonomo

**Affiliations:** 1 "G. Mazzini" Hospital – ASL Teramo, Teramo, Italy; 2 Università Cattolica del Sacro Cuore – Policlinico A. Gemelli, Rome, Italy

**Keywords:** Linear Regression Analysis, Acute Myocardial Infarction, Infarct Size, Acute Myocardial Infarction, Functional Recovery

## Purpose

To evaluate by MRI the effect of G-CSF on ventricular remodeling after AMI treated with PTCA.

## Materials and methods

14 patients with AMI (TIMI 3), 7 tretaed with G-CSF administration (5 mcg/kg sc for 5 days) studied by MRI (GE 1.5 T) within 7 days and after 4–6 months to evaluate end systolic (ESV) and end diastolic (EDV) volumes and ejection ffraction (EF). B-SSFP sequences for functional study, FGRET forFirst Pass study (FP) and IR-prep FGRE for Delayed Enhancement (DE). 0,1 mmol/Kg of Gd-DTPA were administered with a 3 ml/sec flow both for FP and DE (total dose 0,2 mmol/kg). FP and DE were visually scored accounting for transmural and circumferential extent.

FP and DE correlation with functional recovery and differences between groups were evaluated by linear regression analysis and covariance analysis (ANCOVA).

## Results

A statistically significant correlation between DE and functional recovery at follow up is present (for ESV p = 0,05; for EF p = 0,03); there was no significant linear correlation with FP.

ANCOVA showed a positive trend for patients treated with G-CSF, for any infarct size, especially for EDV and ESV (mean G-CSF effect: EDV -29,4 ml, p = 0,045; ESV -28 ml p = 0,06).

## Conclusion

MRI allows to correctly evaluate differences in functional recovery between different groups of patients, accounting for infarct size. We found a positive trend with partial statistical significance regarding global functional recovery in patients treated with G-CSF after AMI. These data need however to be confirmed in larger series, as other studies (REVIVAL2) did not show significant correlations.

